# Identify novel inflammation-related prognostic signature in pancreatic cancer patients

**DOI:** 10.1097/MD.0000000000036932

**Published:** 2024-02-16

**Authors:** Yuan Sun, Xiaoying Zhang, Haiyan Zhu

**Affiliations:** a Department of Gastroenterology, the First Hospital of China Medical University, Shenyang, China; b Central Sterile Supply Department, the First Hospital of China Medical University, Shenyang, China.

**Keywords:** AUTODOCK, GSEA, inflammation, pancreatic cancer, prognostic signature

## Abstract

Pancreatic cancer (PC) is a malignant tumor of the digestive system with a poor prognosis. PC patients with pancreatitis have a worse prognosis. But nobody reported the relationship between inflammation and prognosis in PC. Based on this, we are going to explore inflammation-related prognostic signature to predict patients’ survival and potential therapeutic target. We screened gene expression profile and corresponding clinical information of patients from The Cancer Genome Atlas (TCGA) database. Gene set enrichment analysis (GSEA) was performed to identify differentially expressed genes (DEGs) between tumor and normal tissues with *P* value < .05. Univariate and multivariate Cox regression analysis was applied to identify possible prognostic inflammation genes and establish an inflammation-related risk score system, which was validated by Kaplan–Meier and Receiver operating characteristic (ROC) curves. Finally, we used the TISIDB database to predict targeted drugs for up-regulated gene hepatocyte growth factor receptor (MET) and used AUTODOCK software for molecular docking. We built a prognostic model consisted of 3 inflammation-related genes (tumor necrosis factor receptor associated factor 1/TFAR1, tyrosine kinase 2/TYK2, MET). According to the median value of those genes’ risk score, PC patients were ranked into high- (88) and low-risk (89) groups. Then, the results of the Kaplan–Meier curves and the area under the curve (AUC) of the ROC curves showed this model had a good predictive power (*P* < .001, AUC = 0.806). The result of human protein atlas (HPA) database showed the expression of TRAF1 and TYK2 were low in pancreatic cancer, the expression of MET was high. TISIDB database founded brigatinib could target to MET. And AUTODOCK showed brigatinib had a nice docking with MET. Taken together, our study suggested that inflammation-associated prognostic signature might be used as novel biomarkers for predicting prognosis in PC patients and potential therapeutic target of the disease.

**Highlights**

1. We constructed the inflammation-related prognostic model consisted of TRAF1, TYK2 and MET in PC patients.
2. This model showed a great clinical value in groups of different clinical parameters.
3. We predicted a possible drug (brigatinib) that targeted the MET gene, and had a good docking among them.

## 1. Introduction

Pancreatic cancer (PC) is a highly malignant digestive system cancer with a poor prognosis and a 5-year survival rate of less than 5% following diagnosis.^[1]^ PC was the fourth leading cause of cancer-related deaths in the United States, it was reported that 56,000 new cases and 47,050 deaths for PC patients in 2020.^[2]^ Despite continuous advancements in technical and pharmacological management, the mortality rate of PC remains high. If the condition is not changed, the disease is expected to be the second leading cause of cancer-related mortality in the next decade.^[3,4]^ Therefore, identifying new prognosis biomarkers and therapeutic targets become key points to treat the disease.

Inflammation was essential to the development and progression of the tumor. PC progression resulted from the creation and accumulation of fibroinflammation stroma, which played vital roles in protecting tumor cells from immune-mediated destruction.^[5]^ Besides, chronic inflammation resulted from fibrotic stroma could provide a source of growth factors and proteases that promoted the growth and invasion of pancreatic cancer.^[6]^ Studies have reported that prevalence of PC in patients with pancreatitis was more than 50 times compared with non-pancreatitis, which suggested inflammation was a great possibility to contribute to the development and progression of cancer.^[7]^ Another study showed that the reduction of steroid inflammation in murine models resulted in a significant decrease in tumor development and spread.^[8]^ In conclusion, we hypothesis that inflammation plays a vital role in the development and regulation of pancreatic cancer. However, there have been few systematic studies of inflammation-related genes and their prognostic importance in patients with pancreatic cancer. Based on this, we are going to explore inflammation-related prognostic signature to predict patients’ survival and potential therapeutic target.

## 2. Materials and methods

### 2.1. Data acquisition

Gene expression data (FPKM) and corresponding clinical information of PC patients were acquired from The Cancer Genome Atlas (TCGA, http://cancergenome.nih.gov/). 178 PC patients with the associated gene expression file and complete clinical characteristics were included in our study. The basic clinical features are shown in Table 1.

**Table 1:** Clinical pathological parameters of pancreatic cancer patients.

	TCGA
No. of patients	185
Age (median)	65
Gender (%)	
Female	83 (45)
Male	102 (55)
Grade (%)	
G1–2	129 (69)
G3–4	53 (29)
Unknown	3 (2)
Stage (%)	
Stage I	21 (11)
Stage II	152 (82)
Stage III	4 (2)
Stage IV	5 (3)
Unknown	3 (2)
T (%)	
T1–2	31 (17)
T3–4	152 (82)
Unknown	2 (1)
N (%)	
N0	50 (27)
N1–3	130 (70)
Unknown	5 (3)
M (%)	
M0	85 (46)
M1	5 (3)
Unknown	95 (51)
Survival status	
OS days (median)	547

TCGA = The Cancer Genome Atlas.

### 2.2. Differentially expressed inflammatory genes were identified

Firstly, the expression data set with 56,530 mRNAs was downloaded from the TCGA. Gene set enrichment analysis (GSEA, http://www.broadinstitute.org/gsea/index.jsp) was performed using the above-mentioned data to explore which gene sets were enriched for further analysis.^[9,10]^ Next, we used adjusted *P* value < .05 to identify differentially expressed genes (DEGs) between carcinoma and adjacent tissue. Finally, differentially expressed inflammation-related genes were clearly screened out. And these filtered genes were used to constitute the inflammation-related risk signature for further study. Meanwhile, Gene Ontology (GO) and Kyoto Encyclopedia of Genes and Genomes (KEGG) pathway enrichment analysis were performed for PC patients with *P* value < .05.^[9]^

### 2.3. The inflammation-related risk score system was constructed

We further analyzed the selected genes to construct the inflammation-related prognostic model. Univariate Cox regression analysis was performed to identify possible prognostic genes. Multivariate Cox regression analysis was applied to build prognostic model for PC patients. Then, the risk score for the prognostic model was calculated using the following formula: score = e^sum (each gene’s expression × corresponding coefficient)^. Accordingly, PC patients were classified with risk scores into high- and low-risk groups by using the median value of risk score as the cutoff point.

### 2.4. Assessing the prognosis of inflammation gene signature

Subsequently, Kaplan–Meier (K–M) curves were performed to compare survival rate between high-risk and low-risk groups. Receiver operating characteristic (ROC) curves were drawn according to the risk scores and survival status of each patient to compare the predictive ability of gene signature.^[11]^ Based on the expression of genes signature, principal component analysis (PCA) and t-distributed stochastic neighbor embedding (t-SNE) analysis were applied to explore the distribution of the high-risk and low-risk groups in PC patients. Similarly, univariate and multivariate Cox regression analyses were used to investigate the effects of clinical parameters on prognostic characteristics. Finally, we also mined possible gene mutations in PC patients using TISIDB (http://cis.hku.hk/TISIDB)^[12]^ and visualized by drug-bank (https://www.drugbank.ca/drugs). At the same time, AUTODOCK software was used for molecular docking to further verify the targeted binding effect of drug.

### 2.5. Statistical analysis

Statistical analyses were performed using R (version 4.0.2) software packages. Perl language was used for the data matrix and all data processing. A two-tailed *P* value < .05 was considered statistically significant.

### 2.6. Ethical review statement

This study did not involve the testing of human and animal samples. Images of immunohistochemical staining were derived from the Human Protein Atlas public database and did not require ethics committee approval.

## 3. Results

### 3.1. Five gene sets associated with inflammation were selected

**Figure 1.**
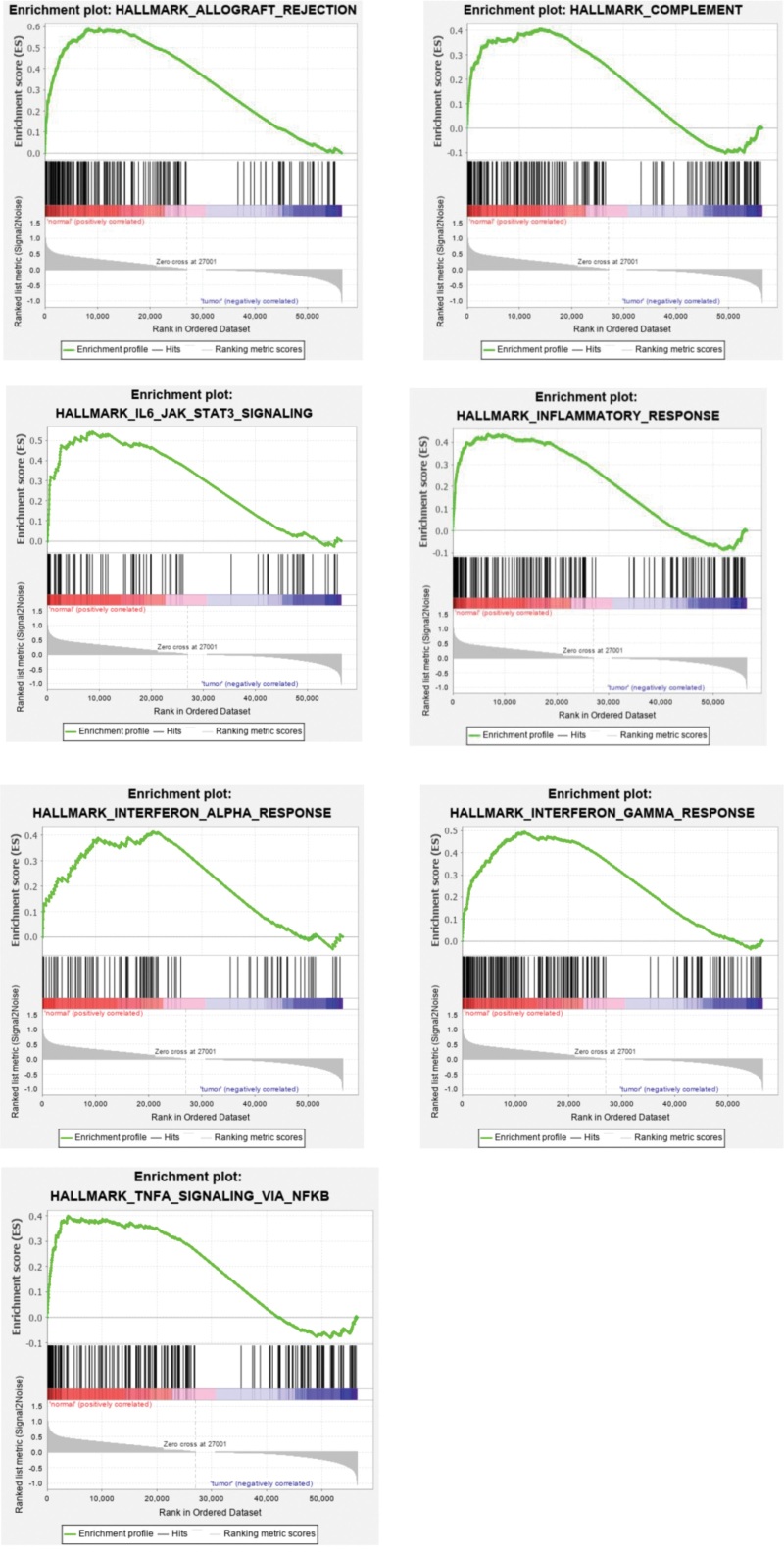
Enrichment plots of gene sets that were related to inflammation between tumor and normal tissue using gene set enrichment analysis.

Initially, the expression data set and the hallmark gene sets were acquired from the TCGA and the Molecular Signatures Database databases. The above data were analyzed by using GSEA to investigate whether the identified gene sets had statistically significant differences between tumor tissues and normal tissues. We found that 7 of the 50 genes were observably enriched with the normalized *P* value < .05 (Table 2; Fig. 1). Then we selected 5 gene sets, containing 341 genes, which were related to inflammation for further analysis (see Table S1, Supplemental Digital Content, http://links.lww.com/MD/L491 which lists 341 genes from 5 gene sets which were related to inflammation for further analysis).

**Table 2:** Selected 7 gene sets which were observably enriched with the normalized *P* value < .05.

Name	Size	ES	FDR q-val	Rank at max
HALLMARK_ALLOGRAFT_REJECTION	200	0.58891	0	10,333
HALLMARK_IL6_JAK_STAT3_SIGNALING	87	0.54276	4.57E-04	8713
HALLMARK_INTERFERON_GAMMA_RESPONSE	200	0.49445	3.04E-04	11,744
HALLMARK_inflammation_RESPONSE	200	0.43642	0.009265896	6824
HALLMARK_COMPLEMENT	200	0.40674	0.03278907	13,892
HALLMARK_TNFA_SIGNALING_VIA_NFKB	200	0.39832	0.03806482	3831
HALLMARK_INTERFERON_ALPHA_RESPONSE	97	0.41374	0.047526434	20,938

### 3.2. Different expression of inflammation genes in PC compared with normal pancreas

**Figure 2.**
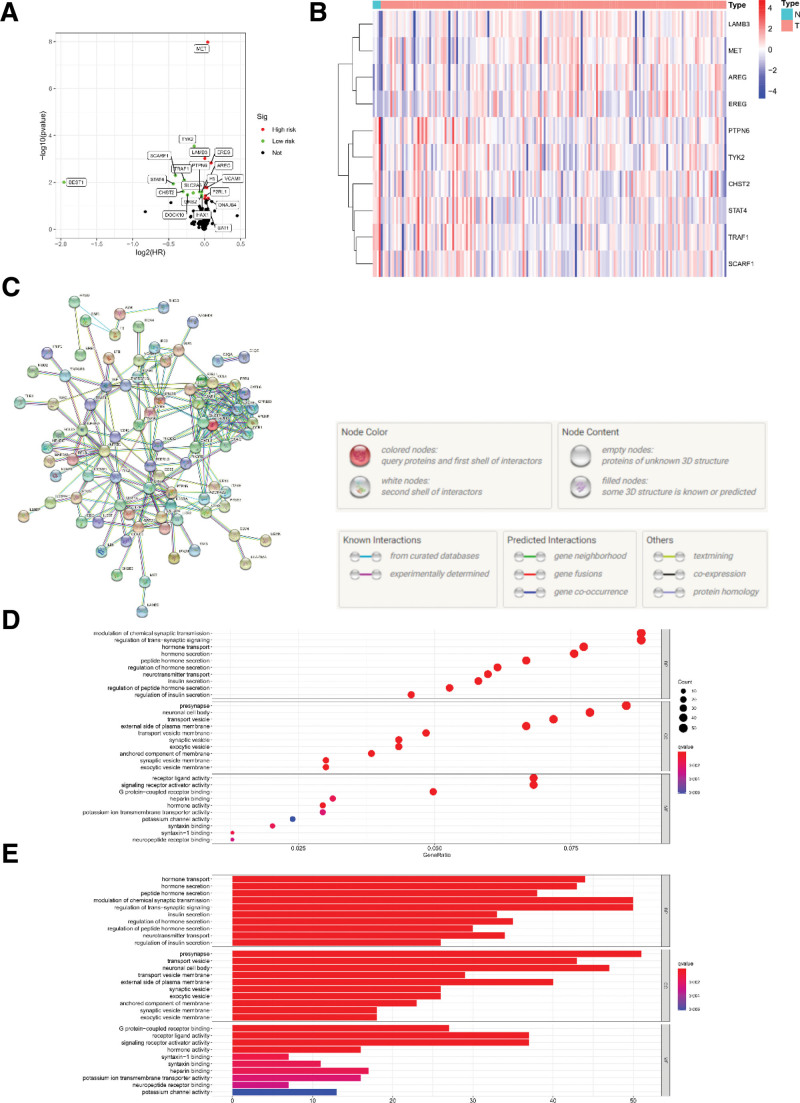
The expression level of differentially expressed genes. (A and B) The result of the volcano plot and heat map showed up-regulated genes (8, 4) and down-regulated genes (12, 6) in DEGs and genes related to inflammation, respectively. (C) Several key genes (C3AR1, NFKB1, CCL4, CXCR6, APLNR) were identified by the PPI network. (D and E). GO (D) and KEGG (E) analysis results of DEGs.

Primarily, the selected genes were visualized by the volcano plot and heat map. We found that those genes consisted of 4 up-regulated genes and 6 down-regulated genes in DEGs and inflammation-related genes (Fig. 2A and B). Next, GO and KEGG were performed to explore the enrichment situation of those genes. We observed that most of them were enriched in Hormone secretion Related function and pathways (Fig. 2C and D). Finally, the protein-protein interaction network was applied to further discover hub genes (C3a receptor-1/C3AR1, nuclear factor kappa B subunit 1/NFKB1, chemokine ligand 4/CCL4, CXC chemokine receptor 6/CXCR6, apelin receptor/APLNR) (Fig. 2E).

### 3.3. Prognostic model was constructed by TRAF1, TYK2, and MET

First of all, univariate Cox regression analyses were carried out to look for possible prognostic genes among selected inflammation-related differential genes. We caught sight of 10 potential genes, including amphiregulin (AREG), signal transducer and activator of transcription 4 (STAT4), protein tyrosine phosphatase nonreceptor type 6 (PTPN6), tyrosine kinase 2 (TYK2), tumor necrosis factor receptor associated factor 1 (TRAF1), scavenger receptor class F member 1 (SCARF1), epiregulin (EREG), GlcNAc6ST-1 (CHST2), laminin subunit beta 3 (LAMB3), hepatocyte growth factor receptor (MET) (Table 3). Multivariate Cox regression analyses found that there 3 inflammation-related genes were a great possibility of building the best prognostic model for PC patients (Table 3). The prognostic signature was constructed to predict prognosis based on the risk score = e ^((−0.1) * expression level of TRAF1 + (−0.1) * expression level of TYK2 + 0.03 * expression level of MET)^. The PC patients were divided into a high-risk group (n = 535) and a low-risk group (n = 535) based on the median value as the cutoff point for further verification.

**Table 3:** Univariate and multivariate cox regression analysis between differentially expressed genes.

Gene	Univariate cox regression analysis				Multivariate cox regression analysis	
	HR	HR.95L	HR.95H	Cox *P* value	Coef.	HR
AREG	1.00693	1.001274	1.012624	0.016277	–	–
STAT4	0.73941	0.585289	0.934106	0.011356	–	–
PTPN6	0.95395	0.915357	0.994164	0.025235	–	–
TYK2	0.9044	0.85662	0.954842	0.000285	−0.067	0.935184
TRAF1	0.82227	0.711531	0.950244	0.008013	−0.111	0.894926
SCARF1	0.7548	0.620185	0.918628	0.005003	–	–
EREG	1.06539	1.024589	1.10782	0.001477	–	–
CHST2	0.81193	0.677436	0.973126	0.024146	–	–
LAMB3	1.00292	1.001187	1.004655	0.000948	–	–
MET	1.03093	1.020232	1.041749	1.05E-08	0.02754	1.02792

HR = hazard ratio.

### 3.4. Predictive capability of the prognostic model was robust

**Figure 3.**
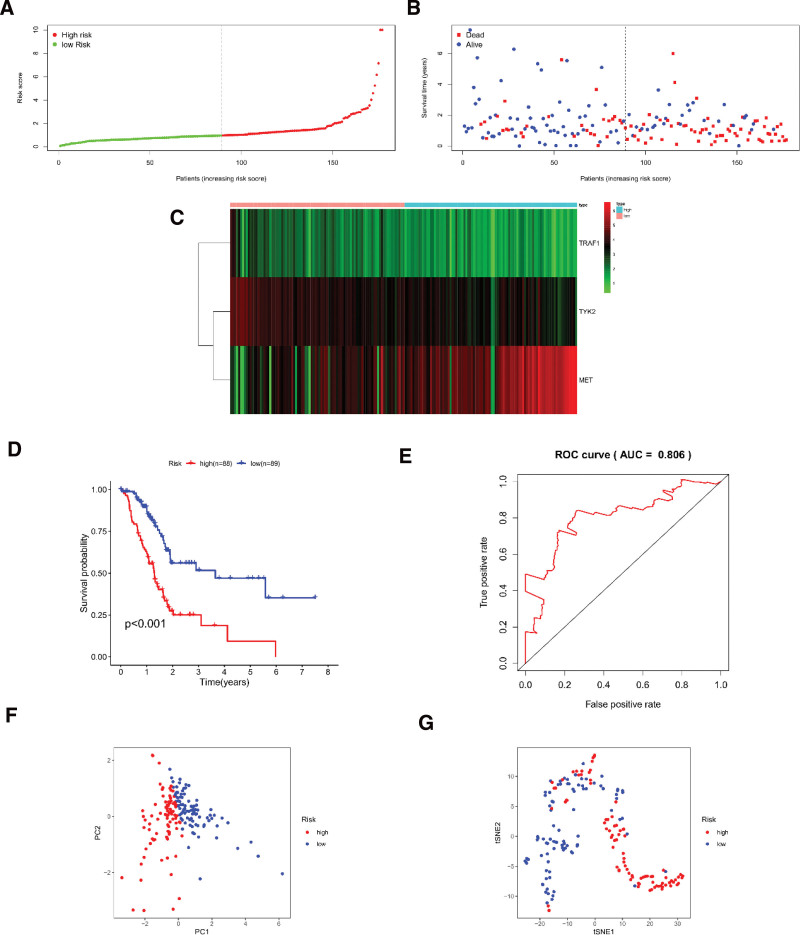
Identification of the candidate inflammation-related genes in TCGA. (A and B) The higher the risk score, the worse the prognosis of PC patients. (C) TRAF1 and TYK2 are lower expressed in PC patients, while MET is higher expressed. (D) K-M survival curve showed a worse prognosis in the high-risk group than in the low-risk group (*P* < .001). (E) AUC of time-dependent ROC curves revealed the model has a robust predictive ability (AUC = 0.806). (F and G) PCA and t-SNE analysis plots were able to divide the high-low risk group into two parts.

According to the results of univariate and multivariate Cox regression analysis, we observed that the prognostic signature could serve as an independent prognostic biomarker to predict patients’ survival outcomes. Meanwhile, we assessed the distribution and median value of the risk scores in Figure 3A–C. To validate the predictive power of the prognostic model, K–M survival analysis were performed in the high- and low-risk group. And the ROC curves were used to assess predictive performance of this prognostic signature in the high- and low-risk group. The K–M survival curve showed that the prognostic model significantly distinguished patients into high and low survival group (*P* < .01; Fig. 3D). The area under the curve (AUC) of ROC curves substantiated that the prognostic signature had a fine capability on predicting the over survival for PC patients (AUCs = 0.806; Fig. 3E). Meanwhile, this condition was observed that survival time was significantly lower in the high-risk group than in the low-risk group. All results demonstrated that this model can provide reliable prognostic information for PC patients. Besides, PCA and t-SNE analysis were performed to distributed PC patients into two directions in different risk groups, which indicated our model possessed a good assessment ability (Fig. 3F–G).

### 3.5. The expression of inflammation-related prognostic signature was higher in PC than in para cancerous tissue

**Figure 4.**
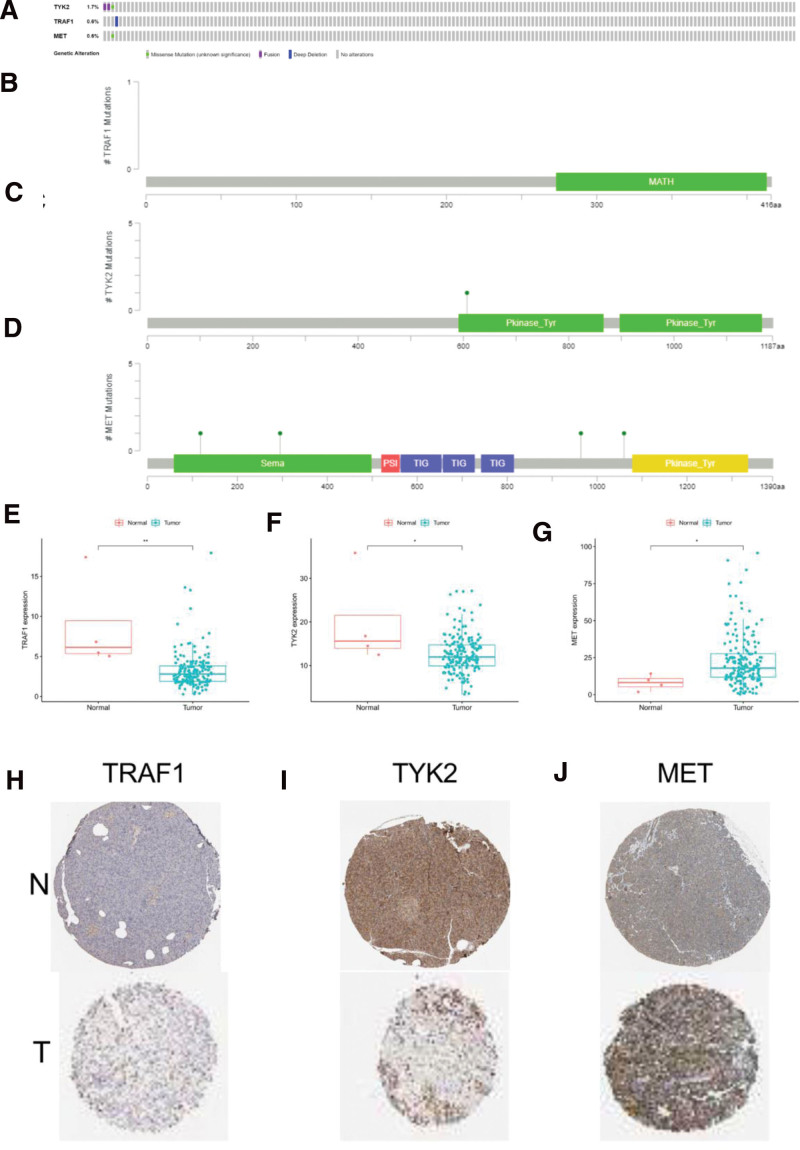
(A) The mutations of TRAF1, TYK2 and MET were 1.6%, 0.6%, and 0.6%, respectively. (B–D). TRAF1, TYK2 and MET specific locations of mutations. (E–J) The expression of TRAF1, TYK2, and MET was displayed by box plots (E–G) and Immunohistochemistry (H–J) in tumor and normal tissue.

Firstly, we investigated the alterations of the 3 selected genes by analyzing 185 PC patients in the cBioPortal database (http://www.cbioportal.org/). The TRAF1 gene had a 1.6% change, the TYK2 and MET genes only had 0.6% changes, respectively (Fig. 4A–D). These 3 genes are relatively stable and un-mutational obviously. Then, we observed that the expression of TRAF1 and TYK2 was lower in the cancer tissues than in the adjacent tissues (Fig. 4E and F), however, the expression level of MET is the opposite (Fig. 4G). Similarly, we further confirmed the expression of them in the carcinoma and adjacent tissues through the human protein atlas database (https://www.proteinatlas.org/) (Fig. 4H–J).

### 3.6. Three inflammation-gene signatures had independent prognostic value for clinical parameters

**Figure 5:**
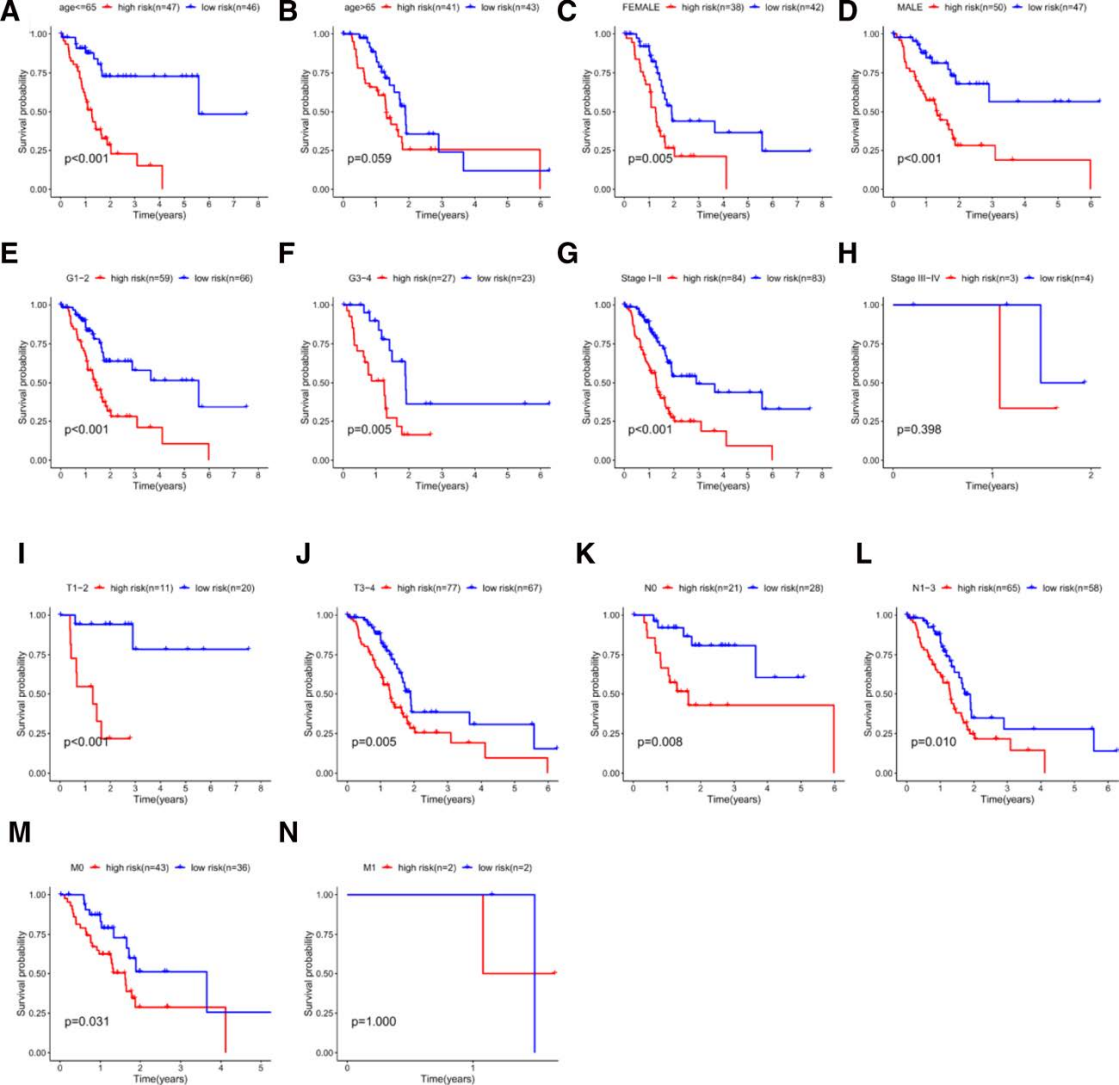
Kaplan–Meier curves for the prognostic signature value of risk score for the patients divided by each clinical feature. (A and B) Age (≤65 or >65). (C and D) Gender (FEMALE or MALE). (E and F) Grade (G1-2 or G3-4). (G and H) Pathological stage (Stage I-II or Stage III-IV). (I and J). Pathological stage-T (T1-2 or T3-4). (K and L) Pathological stage-N (N0 or N1-N3). (M and N) Pathological stage-M (N0 or N1-N3).

Based on the importance of prognosis to doctors and patients, we further performed Cox regression analyses to track the correlation among the prognostic signature and different clinicopathological parameters, including age, gender, pathological grade, pathological state (T, N, M), risk score. Univariate Cox regression analysis showed that pathological stage-T, pathological stage-N and risk score can be prognostic indicators for PC patients (Table 4). Multivariate Cox regression analysis further confirmed that pathological stage-N (*P* = .027) and the risk score (*P* < .001) were independent prognostic biomarkers (Table 4). Considering the difference of individual patient, stratified survival analysis was used to explore whether the risk model can differentiate survival differences between different subgroups. About clinical parameters, age (<=65 or > 65; Fig. 5A and B), gender (FEMALE or MALE; Fig. 5C and D), grade (G1-2 or G3-4; Fig. 5E and F), pathological stage (Stage I-II or Stage III-IV; Fig. 5G and H), pathological stage-T (T1-2 or T3-4; Fig. 5I and J), pathological stage-N (N0 or N1-N3; Fig. 5K and L), pathological stage-M (M0 or M1; Fig. 5M and N), we noticed that the survival time was obviously lower in high-risk patients than in the other group. Therefore, the risk model consisted of 3 inflammation genes could be identified as an independent prognostic factor and have significant clinical value for PC patients.

**Table 4:** Univariate and multivariate cox regression analysis among different clinical parameters.

Parameter	Univariate cox regression analysis				Multivariate cox regression analysis			
	HR	HR.95L	HR.95H	*P* value	HR	HR.95L	HR.95H	*P* value
Age	1.351796	0.8665	2.108891	0.184029	1.212888	0.78896	1.864593	0.379051
Gender	0.713433	0.45394	1.12126	0.143247	0.78136	0.50889	1.199705	0.259438
Grade	1.736349	0.95442	3.158905	0.070737	1.386162	0.88392	2.173766	0.154888
Stage	1.633854	0.44147	6.046754	0.462144	0.888789	0.27998	2.82142	0.841452
T	1.251436	0.59983	2.610894	0.549991	2.032571	1.01356	4.076078	0.045728
N	1.993298	1.08171	3.673115	0.02698	2.257961	1.30802	3.897787	0.003456
M	0.690787	0.32658	1.461148	0.333135	1.060686	0.62146	1.810354	0.828992
Risk score	0.419253	0.2595	0.677364	0.000383	0.404705	0.25671	0.638025	9.83E-05

HR = hazard ratio.

### 3.7. Brigatinib had a great possibility to targeted MET

**Figure 6:**
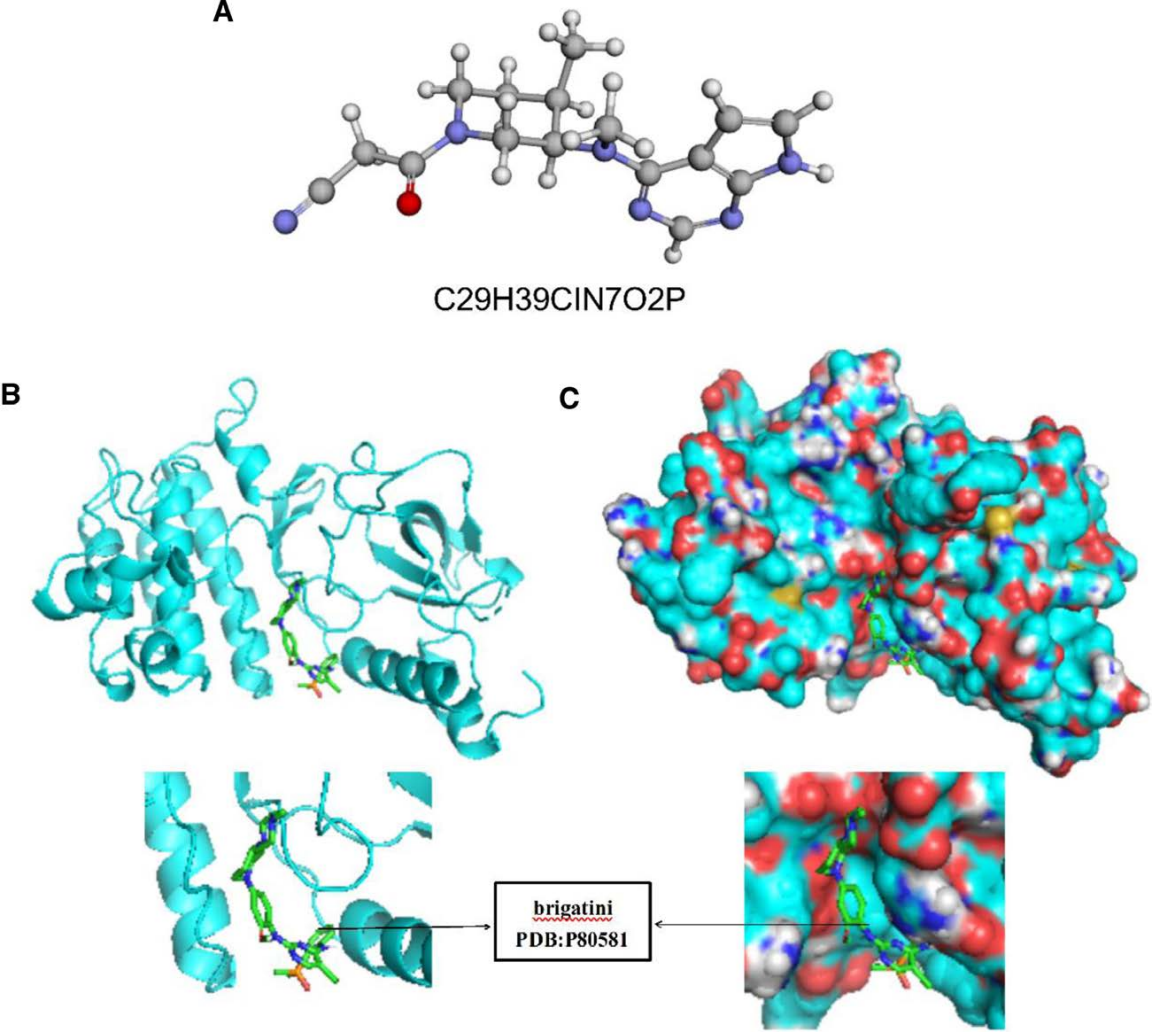
The prediction of targeted drug on prognostic gene signature. (A) The molecular formula and structural formula of brigatinib. (B and C) The Binding site of brigatinib with MET.

Additionally, drug-targeted analysis of the prognostic signature showed that brigatinib might be a potential therapeutic drug for PC patients, as drugs can target MET gene and they have been reported to inhibit inflammation. The result of AUTODOCK also showed that brigatinib had a good targeted binding property with MET protein (Fig. 6A–C). Therefore, the small molecule drug has potential clinical value for improving patients’ prognosis. Then, the prognostic signature identified in the study could serve as a prognostic biomarker for PC patients and would eventually apply to personalized targeted therapy.

## 4. Discussion

At present, the prognosis of pancreatic cancer, known as the king of cancer, is still not ideal due to the initial diagnosis of the terminal stage with metastasis and limitations of therapeutic drugs. Thus, we downloaded the uploaded data from the TCGA database to explore prognostic genes and predict and develop a targeted drug.

Firstly, we utilized the GSEA database to screen inflammation-related gene sets, 5 of which were selected and contained 341 genes. Subsequently, we applied univariate and multivariate regression analyses to identify possible prognostic genes (TRAF1, TYK2, MET). These 3 genes have been reported extensively in the literature. The expression of TRAF1 is low in renal carcinoma cells and primary central nervous system lymphomas. And TRAF1 may have a pro-apoptotic and anti-mitotic effects.^[13,14]^ The researches of reported bioinformatics showed the expression level of TYK2, as one of prognostic biomarkers, was higher in tumor tissues than in normal tissues in breast cancer and hepatocellular carcinoma.^[15,16]^ Besides, MET was also regarded as a potential target across all Papillary Renal Cell Carcinomas.^[17,18]^ Consistently, our results also observed that the expression of TRAF1 and TYK2 were low, the expression of MET was high in pancreatic cancer.

Therefore, we used the 3 genes as risk models to predict the prognosis of pancreatic cancer patients. The feasibility and sensitivity of the model are verified from 3 aspects. Primarily, the expression of the 3 genes in tumor and normal tissue was consistent with the prognosis of patients with pancreatic cancer. Secondly, the K-M curve manifested a *P* < .001, and the AUC of the ROC curve was 0.806. And then, PCA and t-SNE divided pancreatic cancer patients into two components with high and low-risk groups based on a median value. Thirdly, the model had a good prediction ability for the overall characteristics of clinical parameters and the survival rate of each group. Fourthly, the experimental result of cell lines offered a favorable evidence for the reliability of the model (*P* < .05).

Gemcitabine monotherapy or gemcitabine-based combination therapy, has been the standard systemic therapy for advanced pancreatic cancer,^[19,20]^ but drug resistance was also common. Therefore, we performed TISIDB and the drug-Bank database to predict a novel targeted drug (brigatinib) for MET genes identified as prognostic signatures. According to many investigations, brigatinib was an effective and safe targeted drug for advanced ALK-positive non-small-cell lung cancer^[21–23]^ And the result of AUTODOCK showed brigatinib had a nice docking with MET. Based on the above description, we speculated brigatinib might offer a great possibility for the therapeutic regimen of patients with pancreatic cancer.

## 5. Conclusion

In conclusion, this study provided a good model to predict patient prognosis. Meanwhile, it has been validated in many aspects, and possible targeted therapeutic drug have been proposed.

## Author contributions

**Conceptualization:** Yuan Sun.

**Data curation:** Yuan Sun, Xiaoying Zhang.

**Formal analysis:** Yuan Sun.

**Investigation:** Yuan Sun, Xiaoying Zhang.

**Methodology:** Xiaoying Zhang.

**Resources:** Xiaoying Zhang.

**Supervision:** Haiyan Zhu.

**Validation:** Haiyan Zhu.

**Writing – original draft:** Yuan Sun.

**Writing – review & editing:** Haiyan Zhu.

## Supplementary Material


