# Supplementary material for: Identify novel inflammation-related prognostic signature in pancreatic cancer patients
Source: Medicine (Baltimore). 2024 Feb 16;103(7):e36932. doi: 10.1097/MD.0000000000036932 (PMC10869063; doi:10.1097/MD.0000000000036932)
Supplement: Supplementary file 1 [file medi-103-e36932-s001.docx]

**Table S1 341 genes from 5 gene sets which were related to inflammation for further analysis.**

| A2M |
| --- |
| ACVR1B |
| ACVRL1 |
| BAK1 |
| CBL |
| CCL7 |
| CCR1 |
| CD14 |
| CD36 |
| CD38 |
| CD44 |
| CD9 |
| CNTFR |
| CRLF2 |
| CSF1 |
| CSF2 |
| CSF2RA |
| CSF2RB |
| CSF3R |
| CXCL1 |
| CXCL10 |
| CXCL11 |
| CXCL13 |
| CXCL3 |
| CXCL9 |
| DNTT |
| EBI3 |
| FAS |
| GRB2 |
| HAX1 |
| HMOX1 |
| IFNAR1 |
| IFNGR1 |
| IFNGR2 |
| IL10RB |
| IL12RB1 |
| IL13RA1 |
| IL15RA |
| IL17RA |
| IL17RB |
| IL18R1 |
| IL1B |
| IL1R1 |
| IL1R2 |
| IL2RA |
| IL2RG |
| IL3RA |
| IL4R |
| IL6 |
| IL6ST |
| IL7 |
| IL9R |
| INHBE |
| IRF1 |
| IRF9 |
| ITGA4 |
| ITGB3 |
| JUN |
| LEPR |
| LTB |
| LTBR |
| MAP3K8 |
| MYD88 |
| OSMR |
| PDGFC |
| PF4 |
| PIK3R5 |
| PIM1 |
| PLA2G2A |
| PTPN1 |
| PTPN11 |
| PTPN2 |
| REG1A |
| SOCS1 |
| SOCS3 |
| STAM2 |
| STAT1 |
| STAT2 |
| STAT3 |
| TGFB1 |
| TLR2 |
| TNF |
| TNFRSF12A |
| TNFRSF1A |
| TNFRSF1B |
| TNFRSF21 |
| TYK2 |
| ABCA1 |
| ABI1 |
| ACVR2A |
| ADGRE1 |
| ADM |
| ADORA2B |
| ADRM1 |
| AHR |
| APLNR |
| AQP9 |
| ATP2A2 |
| ATP2B1 |
| ATP2C1 |
| AXL |
| BDKRB1 |
| BEST1 |
| BST2 |
| BTG2 |
| C3AR1 |
| C5AR1 |
| CALCRL |
| CCL17 |
| CCL2 |
| CCL20 |
| CCL22 |
| CCL24 |
| CCL5 |
| CCR7 |
| CCRL2 |
| CD40 |
| CD48 |
| CD55 |
| CD69 |
| CD70 |
| CD82 |
| CDKN1A |
| CHST2 |
| CLEC5A |
| CMKLR1 |
| CSF3 |
| CX3CL1 |
| CXCL6 |
| CXCL8 |
| CXCR6 |
| CYBB |
| DCBLD2 |
| EDN1 |
| EIF2AK2 |
| EMP3 |
| EREG |
| F3 |
| FFAR2 |
| FPR1 |
| FZD5 |
| GABBR1 |
| GCH1 |
| GNA15 |
| GNAI3 |
| GP1BA |
| GPC3 |
| GPR132 |
| GPR183 |
| HAS2 |
| HBEGF |
| HIF1A |
| HPN |
| HRH1 |
| ICAM1 |
| ICAM4 |
| ICOSLG |
| IFITM1 |
| IL10 |
| IL10RA |
| IL12B |
| IL15 |
| IL18 |
| IL18RAP |
| IL1A |
| IL2RB |
| IL7R |
| INHBA |
| IRAK2 |
| IRF7 |
| ITGA5 |
| ITGB8 |
| KCNA3 |
| KCNJ2 |
| KCNMB2 |
| KIF1B |
| KLF6 |
| LAMP3 |
| LCK |
| LCP2 |
| LDLR |
| LIF |
| LPAR1 |
| LTA |
| LY6E |
| LYN |
| MARCO |
| MEFV |
| MEP1A |
| MET |
| MMP14 |
| MSR1 |
| MXD1 |
| MYC |
| NAMPT |
| NDP |
| NFKB1 |
| NFKBIA |
| NLRP3 |
| NMI |
| NMUR1 |
| NOD2 |
| NPFFR2 |
| OLR1 |
| OPRK1 |
| OSM |
| P2RX4 |
| P2RX7 |
| P2RY2 |
| PCDH7 |
| PDE4B |
| PDPN |
| PLAUR |
| PROK2 |
| PSEN1 |
| PTAFR |
| PTGER2 |
| PTGER4 |
| PTGIR |
| PTPRE |
| PVR |
| RAF1 |
| RASGRP1 |
| RELA |
| RGS1 |
| RGS16 |
| RHOG |
| RIPK2 |
| RNF144B |
| ROS1 |
| RTP4 |
| SCARF1 |
| SCN1B |
| SELE |
| SELENOS |
| SELL |
| SEMA4D |
| SERPINE1 |
| SGMS2 |
| SLAMF1 |
| SLC11A2 |
| SLC1A2 |
| SLC28A2 |
| SLC31A1 |
| SLC31A2 |
| SLC4A4 |
| SLC7A1 |
| SLC7A2 |
| SPHK1 |
| SRI |
| STAB1 |
| TACR1 |
| TACR3 |
| TAPBP |
| TIMP1 |
| TLR1 |
| TLR3 |
| TNFAIP6 |
| TNFRSF9 |
| TNFSF10 |
| TNFSF15 |
| TNFSF9 |
| TPBG |
| VIP |
| ADAR |
| B2M |
| BATF2 |
| C1S |
| CASP1 |
| CASP8 |
| CD47 |
| CD74 |
| CMPK2 |
| CMTR1 |
| CNP |
| DDX60 |
| DHX58 |
| ELF1 |
| EPSTI1 |
| GBP2 |
| GBP4 |
| GMPR |
| HELZ2 |
| HERC6 |
| HLA-C |
| IFI27 |
| IFI30 |
| IFI35 |
| IFI44 |
| IFI44L |
| IFIH1 |
| IFIT2 |
| IFIT3 |
| IFITM2 |
| IFITM3 |
| IRF2 |
| ISG15 |
| ISG20 |
| LAP3 |
| LGALS3BP |
| LPAR6 |
| MOV10 |
| MVB12A |
| MX1 |
| NCOA7 |
| NUB1 |
| OAS1 |
| OASL |
| OGFR |
| PARP12 |
| PARP14 |
| PARP9 |
| PLSCR1 |
| PNPT1 |
| PROCR |
| PSMA3 |
| PSMB8 |
| PSMB9 |
| PSME1 |
| PSME2 |
| RNF31 |
| RSAD2 |
| SAMD9 |
| SAMD9L |
| SLC25A28 |
| SP110 |
| TAP1 |
| TDRD7 |
| TENT5A |
| TMEM140 |
| TRAFD1 |
| TRIM14 |
| TRIM21 |
| TRIM25 |
| TRIM26 |
| TRIM5 |
| TXNIP |
| UBA7 |
| UBE2L6 |
| USP18 |
| WARS1 |
